# Inhibitory Effect of Cudratrixanthone U on RANKL-Induced Osteoclast Differentiation and Function in Macrophages and BMM Cells

**DOI:** 10.3389/fphar.2020.01048

**Published:** 2020-08-05

**Authors:** Eun-Nam Kim, Jaeyoung Kwon, Hyun-Su Lee, Sooyeun Lee, Dongho Lee, Gil-Saeng Jeong

**Affiliations:** ^1^ College of Pharmacy, Keimyung University, Daegu, South Korea; ^2^ Natural Constituents Research Center, Korea Institute of Science and Technology (KIST), Gangneung, South Korea; ^3^ Department of Biosystems and Biotechnology, College of Life Sciences and Biotechnology, Korea University, Seoul, South Korea

**Keywords:** Cudratrixanthone U, TRAF6, TAK-1, RANKL, CCL4, osteoclast

## Abstract

Cudratrixanthone U (CTU) is a prenylated xanthone compound isolated from *Maclura tricuspidata* Bureau (Moraceae). Prenylated xanthones have been reported to exhibit a variety of biological activities. However, the effects of prenylated xanthone on osteoclast differentiation and function are still unclear. Excessive bone resorption by osteoclasts is considered a major cause of diseases such as osteoporosis. Accordingly, suppression of excessive osteoclast formation and function is one of strategies for treating osteoclast related bone diseases. In this study, CTU inhibited osteoclast differentiation and function in RAW264.7 macrophages and BMM cells induced by receptor activator of nuclear factor-κB ligand (RANKL). CTU regulated the formation of TRAF6-TAK1 complex in RANKL-induced RAW264.7 macrophages and BMM cells. Osteoclast-specific genes including those encoding matrix metallopeptidase 9 (MMP-9), dendritic cell-specific transmembrane proteins (DC-STAMP), cathepsin K (CTSK) and chemokine CC motif ligand 4 (CCL4) play an important role in bone resorption and migration, and were effectively regulated by CTU. These results suggest that CTU is a potential therapeutic agent in osteoporosis.

## Introduction

Bone homeostasis is associated with a balance between bone resorption by osteoclasts and bone formation by osteoblasts. Bone resorption by excessive osteoclast differentiation can lead to disorders such as osteoporosis and rheumatoid arthritis (Boyce et al., 2005; Warren et al., 2015). Therefore, inhibition of bone resorption by excessive osteoclast differentiation plays a key role in the treatment of osteoporosis. Osteoclasts are giant multinucleated cells derived from the mononuclear macrophage family *via* continual proliferation, differentiation and fusion of hematopoietic stem cells. They are involved in bone resorption, and play an essential role in bone formation and control of bone density (Novack and Teitelbaum, 2008; Geng et al., 2017).

Osteoclast formation requires specific activation of the RANKL/RANK (receptor activator of NF-κB ligand and its receptor) system of macrophage or monocyte lineage. These osteoclasts play an essential role in various diseases (Teitelbaum, 2000). Among the TNF receptor-related factors (TRAF) molecules, TRAF6 is an important component of the RANK signaling pathway. When RANKL binds to RANK in preosteoclasts, TRAFs 2, 3, 5, and 6 are recruited, which induces mitogen-activated protein kinase (MAPK) and nuclear factor kappa-light-chain-enhancer of activated B cells (NF-κB) pathways (Kim et al., 1999), and the activation of various signaling pathways. In addition, RANKL stimulation not only activates transforming growth factor beta-activated kinase 1 (TAK1), which is a member of the mitogen-activated protein kinase (MAPK) family, but also activates the TRAF6-TAK1 complex associated with RANK to induce both c-Jun N-terminal kinase (JNK) and NF-kB activation and downstream signaling pathway activator protein 1 (AP-1), and c-Fos/c-Jun dimer induces the expression of primary osteoclast regulatory nuclear factor of activated T cells c1 (NFATc1) (Takayanagi et al., 2002; Kanzaki et al., 2017). Thus, inhibition of these signaling pathways prevents pathological bone loss induced by excessive osteoclast formation. Activated NFATc1 regulates a variety of genes, such as tartrate-resistant acid phosphatase (TRAP), cathepsin K (CTSK), and dendritic cell-specific transmembrane proteins (DC-STAMP), which are essential for osteoclast differentiation and function (Li et al., 2006). Also, the matrix metalloproteinase (MMP)-9 and integrin β3 secreted by osteoclasts are known to promote osteoclast migration and mediate osteoclast bone resorption (Ishibashi et al., 2006). Previous studies have suggested that the inhibition of osteoclast migration is a potential therapeutic target in bone disease, including osteoporosis, and the chemokine CC motif ligand 4 (CCL4) is known to promote the migration and viability of pre-osteoclast cells (Lee et al., 2018). Therefore, inhibition of these genes may play an important therapeutic role in osteoporosis in order to disrupt the differentiation and function of activated osteoclasts.

*Maclura tricuspidata* Bureau (Moraceae) is a traditional medicinal plant native to Northeast Asia that has long been used for lumbago, hemoptysis, and hematemesis (Jung and Shin, 1990; Chang et al., 2008). In particular, *M. tricuspidata* root is known as “Chuan-po-shi” in traditional Chinese medicine and has been used to treat rheumatism and lumbago, and is one of the most common traditional remedies for cancer in Korea (Kim et al., 2009; Xin et al., 2017). Previous studies have reported that the roots and fruits of *M. tricuspidata* showed various pharmacological activities, such as anti-inflammatory effects (Jeong et al., 2010), cytotoxicity inhibition (Lee et al., 2005), and hepatoprotection (Tian et al., 2005). The major constituents of *M. tricuspidata* include prenylated xanthones and flavonoids, alkaloids, and organic acids (Xin et al., 2017; Li et al., 2018). Prenylated xanthones isolated from *M. tricuspidata* have been reported to inhibit neurotoxicity (Jeong et al., 2008), and exhibit neuroprotective (Kwon et al., 2014), and anti-cancer effects (Seo et al., 2001). Despite these pharmacological activities, studies on bone diseases are still incomplete. Therefore, this study investigated the effect of cudratrixanthone U isolated from *M. tricuspidata* on osteoclast differentiation, migration and bone resorption in RANKL-stimulated RAW264.7 and bone marrow-derived macrophages (BMM) cells.

## Materials and Methods

### Chemicals and Reagents

Minimum Essential Medium Eagle—Alpha Modification (Alpha MEM), fetal bovine serum (FBS), penicillin, and streptomycin were purchased from Welgene Inc. (Korea). Mouse RANKL was purchased from PeproTech (Rocky Hill, NJ, USA). 3-(4,5-Dimethylthiazol-2-yl)-2,5-diphenyltetrazoliumbromide (MTT) was obtained from Amresco Inc. (Solon, OH, USA). Fast Red-violet LB salt, naphthol AS-MX phosphate 4-6-diamidino-2-phenylindole (DAPI) and ρ-nitro-phenylphosphate were obtained from Sigma-Aldrich Fine Chemicals (St. Louis, MO, USA). Antibodies against p-ERK, ERK, p-p38, p38, p-JNK, JNK, NFATc1, c-Fos, integrin β3, MMP-9, CTSK and β-actin were purchased from Cell Signaling Technology (Danvers, MA, USA). Anti-TRAF6, anti-TAK1, IκB-α, p-IκB-α, and NF-κB p65 were obtained from Santa Cruz Biotechnology (Santa Cruz, CA, USA). Also, Antibodies of DC-STAMP and CTSK were obtained Abcam (Cambridge, UK). Secondary antibodies were purchased from Santa Cruz and Cell Signaling Technology. Nuclear and cytoplasmic extraction reagent kit and enhanced chemilu-minescence kit were provided by Pierce Biotechnology (Rockford, IL, USA). Protease inhibitors were purchased from Roche (Hoffmann, NC, USA). Alexa fluor-488 palloidin was acquired from Invitrogen (Waltham, MA, USA). Osteo assay surface multiwell plates were obtained from Corning Inc. (NY, USA). Radioimmunoprecipitation (RIPA) assay buffer and protease and phosphatase inhibitor cocktail were purchased from Fisher Scientific Inc. (Waltham, MA, USA).

### Plant Materials and Isolation of Compounds

A voucher specimen (accession number KH1-4-090814) was deposited at the Department of Biosystems and Biotechnology, Korea University, Seoul, Korea. We isolated cudratrixanthone U (CTU) from the bark of *M. tricuspidata*. CTU was extracted as reported previously and structurally identified using nuclear magnetic resonance (NMR) and high-resolution electrospray ionization mass spectrometry (HRESIMS) (Kwon et al., 2016).

### Cell Culture and Viability Assays

RAW 264.7 and BMM cells (ATCC, Manassas, VA, USA) were cultured in alpha-MEM supplemented with 10% heat-inactivated FBS, 2 mM L-glutamine, and 100 U/ml penicillin/streptomycin. Incubations were carried out at 37°C in 5% CO_2_. We performed an MTT assay following the manufacturer’s instructions to detect CTU cytotoxicity. RAW264.7 and BMM cells were seeded on a 96-well plate at a concentration of 5 × 10^3^ cells/ml, followed by treatment with different concentrations and incubation for 120 h. The concentration of MTT solution was 50 mg/ml, and the absorbance at 490 nm was measured by an ELISA plate reader (Männedorf, Swiss).

### Osteoclast Differentiation

RAW 264.7 and BMM cells were seeded on 24-well plates (5 × 10^3^ cells/well) with complete alpha-MEM containing RANKL (50 ng/ml) in the presence of CTU for 5 days at 37°C and 5% CO_2_.

### Tartrate-Resistant Acid Phosphatase (TRAP) Staining and Activity

After differentiation and osteoclast formation as described above, cells were washed and fixed with 4% paraformaldehyde for 10 min, permeabilized with 0.1% Triton X-100, and finally stained for TRAP with the Leukocyte Acid Phosphatase Kit (Sigma, Cat. No. 387A-1KT). Fixed cells were assayed for tartrate-resistant acid phosphatase (TRAP) activity, according to the manufacturer’s instructions (Saint Louis, MO, USA).

### Actin Ring and DAPI Staining

RAW264.7 and BMM cells were cultured in alpha-MEM, with or without CTU for 5 days. The cells were washed three times with phosphate-buffered saline (PBS) and fixed in 4% formalin for 15 min. The cells were then stained with fluorescein isothiocyanate (FITC)-phalloidin solution for 1 h, and the nuclei were sequentially stained with 2.5 μg/ml 4′,6-diamidino-2-phenylindole (DAPI) solution for 10 min. The images were captured using a fluorescence Olympus IX microscope 71-F3 2PH (Tokyo, Japan).

### Bone Resorption Assay

RAW264.7 and BMM cells were seeded with alpha-MEM containing RANKL (50 ng/ml) in the presence of CTU (0.5, 1, 2, 5 μm) into the wells of an osteo assay surface well for 12 days at 37°C and 5% CO_2_. After 10 days, RAW 264.7 cells were removed by 5% sodium hypochlorite (Saint Louis, MO, USA) treatment and resorption pits were visualized under a light Olympus IX microscope 71-F3 2PH. The resorption area was calculated and analyzed using Image-J software Version 1.52i (USA).

### Cell Migration Assay

RAW264.7 and BMM cells (1.5 × 10^4^ cells/well) were incubated in a 24-well plate for 24 h, followed by constant scratching. Next, cells were treated with α-MEM and 10% FBS with or without RANKL (50 ng/ml) and CTU, and the cells migrating for 5 days were counted with Incucyte^®^ Live-Cell analysis systems (Göttingen, Germany).

### Quantitative Real-Time Polymerase Chain Reaction (qRT-PCR)

Total RNA was extracted from the cells using TRIzol reagent (Bioneer, Korea) according to the manufacturer’s instructions. PCR reaction conditions included 1 μl primer, 11 μl nuclease-free ultrapure water, 4 μl 5× reaction buffer, 1 μl RiboLock RNA enzyme inhibitor (20 U/μl), 2 μl 10mM dNTP mix, and 1 μl RevertAid M-MuLV reverse transcriptase (200 U/μl) (Thermo Fisher Scientific, USA). The cycling conditions were 40 cycles at 50°C for 2 min, 95°C initial denaturation for 10 min, 95°C denaturation for 15 s, and 60°C annealing for 30 s. The primer sequences used are listed in **Table 1**.

**Table 1:** Primer sequences.

Target gene	Sequence	
DC-STAMP	Forward (5’-3’)	GCTGTATCGGCTCATCTCCT
	Reverse (3’-5’)	AAGGCAGAATCATGGACGAC
ATP6v0d2	Forward (5’-3’)	AAGCCTTTGTTTGACGCTGT
	Reverse (3’-5’)	TTCGATGCCTCTGTGAGATG
Acp5	Forward (5’-3’)	CACTCCCACCCTGAGATTTGT
	Reverse (3’-5’)	AAGTAGTGCAGCCCGGAGTA
CTSK	Forward (5’-3’)	TCCGCAATCCTTACCGAATA
	Reverse (3’-5’)	AACTTGAACACCCACATCCTG
MMP-9	Forward (5’-3’)	GATGCGTGGAGAGTCGAAAT
	Reverse (3’-5’)	CACCAAACTGGATGACGATG
CCL4	Forward (5’-3’)	CTTCTGCGATTCAGTGCTGTCA
	Reverse (3’-5’)	GCAAAGGCTGCTGGTCTCATAGTAA
GAPDH	Forward (5’-3’)	ACCCAGAAGACTGTGGATGG
	Reverse (3’-5’)	CACATTGGGGGTAGGAACAC

### Cytosolic and Nuclear Protein Extraction

RAW 264.7 and BMM cells were seeded on culture dishes at a density of 2.5 × 10^5^ cells/dish and treated with indicated concentrations of CTU (0.5, 1, 2, and 5 μM), stimulated with RANKL (50 ng/ml), incubated at 37°C with 5% CO_2_, and then lysed using the RIPA buffer. We prepared the cytosolic and nuclear extracts using an NE-PER nuclear and cytoplasmic extraction reagents kit, according to the manufacturer’s instructions.

### Co-Immunoprecipitation (Co-IP)

RAW264.7 and BMM cells treated with or without RANKL in the presence or absence of CTU (2, 5 μM) was digested using RIPA buffer and centrifuged for 30 min. First, the antibody for the targets TRAF6 and TRAF6-specific IgG was incubated with the Thermo dynabead protein G kit in a tube for 10 min, and the antibody was washed away by placing the tube in a Dynamagnet and removing the supernatant. For immunoprecipitation, cell lysates extracted were incubated with anti-TRAF6 or anti-TAK1 antibody for 1 h at 4°C, followed by 1 h incubation at 4°C with the Thermo Fisher Scientific (Waltham, MA, USA) dynabeads protein G kit, following the manufacturer’s instructions.

### Western Blot Analysis

RAW 264.7 and BMM cells were lysed in RIPA buffer containing protease inhibitors and centrifuged at 14,000 rpm for 30 min. Protein concentration was measured by Bradford assay using a Bio-Rad Bradford assay reagent (Hercules, CA, USA). Amounts of each lysate were separated by sodium dodecyl sulfate polyacrylamide gel electrophoresis (SDS-PAGE). After electrophoresis, proteins were transferred using polyvinylidene difluoride (PVDF) membranes (Hercules, CA, USA). After blocking with TBS-T buffer containing skim milk (5%), we incubated with the primary antibody overnight at 4°C. It was washed, incubated with a secondary antibody (horseradish peroxidase-conjugated anti-rabbit IgG and anti-mouse IgG), and detected with Healthcare Life Science ECL-plus (Tokyo, Japan). Immunoreactive bands were analyzed by LAS 4000 (GE Healthcare Life Science, Tokyo, Japan).

### Statistical Analysis

Each experiment was performed in triplicate and expressed as mean value and standard deviation. Statistical analysis was conducted using SPSS Statistics 19.0 software, and correlations were considered significant at *p <*0.05.

## Results

### Inhibitory Effect of CTU on RANKL-Induced Osteoclast Formation

**Figure 1:**
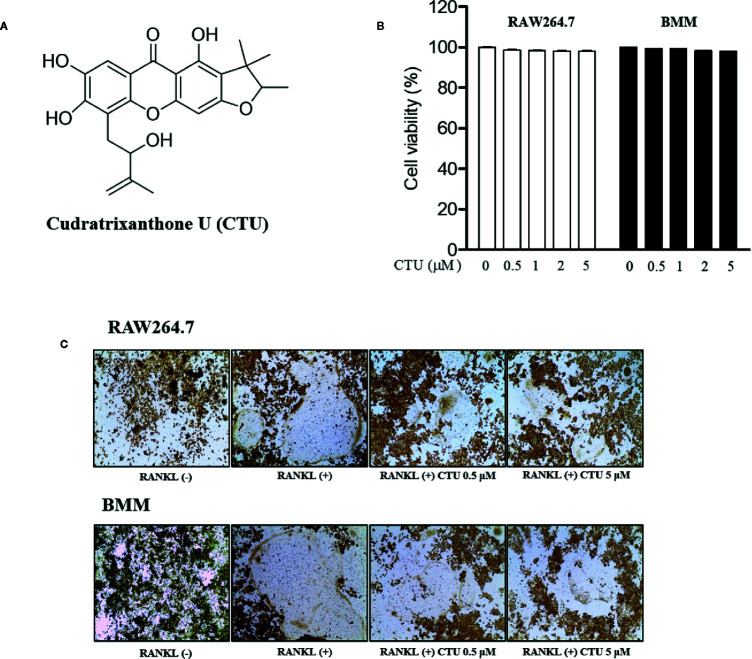
The viability of CTU in RAW264.7 and BMM cells. The chemical structure of cudratrixanthone U (CTU) **(A)**. Effect of CTU on cell viability measured by MTT assay. RAW 264.7 and BMM cells were incubated with various concentrations of CTU for 5 days **(B)**. The effect of CTU in differentiated osteoclasts on morphology **(C)**.

**Figure 2:**
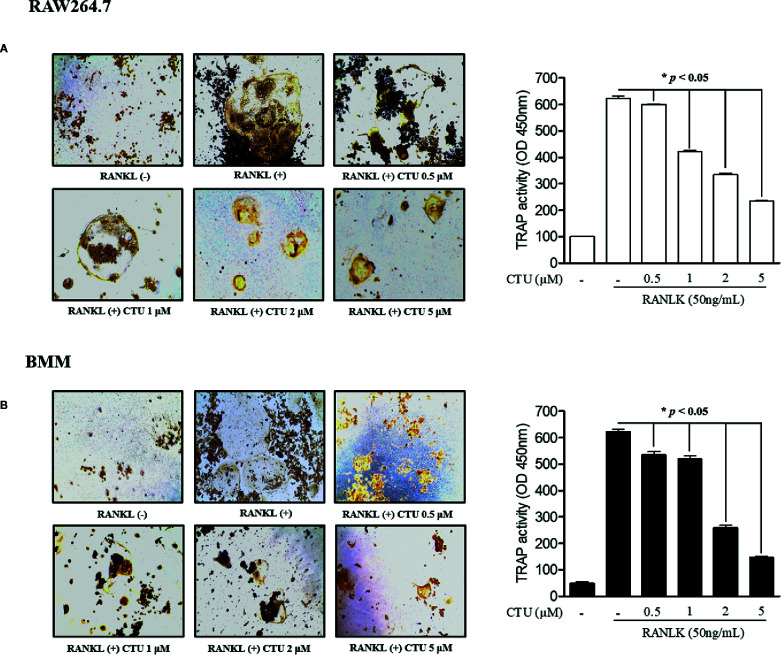
The effect of CTU on osteoclast differentiation in RANKL-induced RAW 264.7 **(A)** and BMM **(B)** cells. The cells were incubated with 50 ng/ml RANKL or both RANKL and various concentrations of CTU for 5 days and then stained for TRAP. TRAP activity was measured using an ELISA reader (optical density, 405 nm). *p < 0.05 compared with the only RANKL-treated group.

To measure CTU (**Figure 1A**) cell viability, we treated RAW264.7 and BMM cells with various concentrations of CTU (0.5, 1, 2, and 5 μM) for 5 days. As shown in **Figure 1B**, there was no significant cytotoxicity due to CTU, also it was confirmed that the morphology of osteoclasts differentiated by RANKL was also inhibited by CTU (**Figure 1C**). Therefore, to verify the effect of CTU on osteoclast formation at the indicated concentrations, we treated CTU with RANKL at different concentrations in RAW264.7 and BMM cells. After 5 days of culture, we measured TRAP staining and activity of mature osteoclasts. Osteoclast differentiation and activity were significantly inhibited by CTU treatment in a dose-dependent manner (**Figures 2A, B**).

### CTU Suppresses Formation of RANKL-Stimulated Osteoclastic F-actin Rings

**Figure 3:**
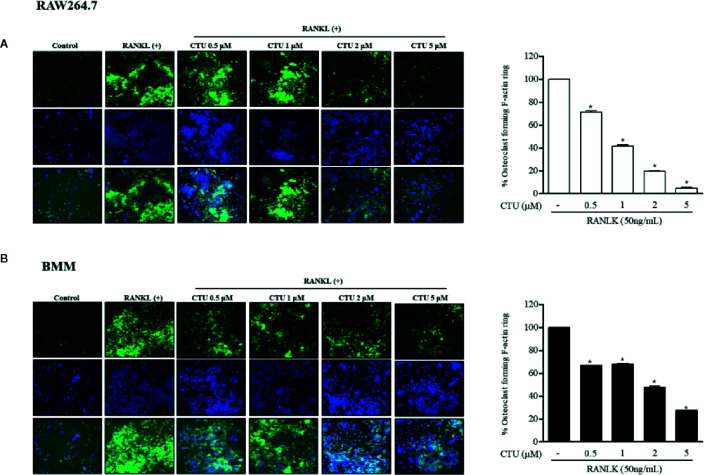
The effect of CTU on osteoclast-actin ring structures in RANKL-induced RAW 264.7 **(A)** and BMM **(B)** cells. RAW 264.7 cells were treated with different concentrations of CTU in the presence of RANKL. After fixation and incubation with Alexa Fluor 488-conjugated phalloidin stained with DAPI, cells were visualized under a ﬂuorescence microscope. Quantitative analysis shows percentage of osteoclasts forming F-actin rings. *p < 0.05 compared with the only RANKL-treated group.

The F-actin ring is a characteristic structure of mature osteoclasts, and therefore we measured the area *via* fluorescence staining of mature osteoclasts to investigate the effect of CTU on RANKL-induced osteoclasts. We found that the formation of the F-actin ring and the pit area were significantly inhibited by CTU in a dose-dependent manner (**Figures 3A, B**). Increased F-actin ring formation by RANKL treatment was concentration-dependently reduced by CTU in RAW264.7 cells and In BMM cells, CTU 0.5 μM and 1 μM treatment group showed similar level of inhibition, and 5 μM treatment group significantly reduced the pit area.

### CTU Inhibited Osteoclast Differentiation and Function

**Figure 4:**
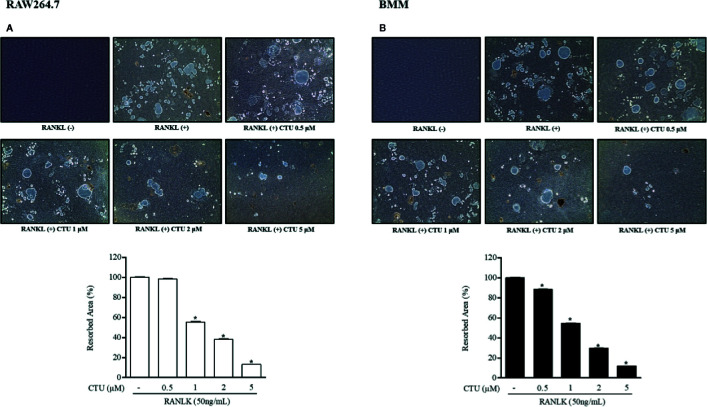
The effect of CTU on osteoclast bone resorption in RANKL-induced RAW 264.7 **(A)** and BMM **(B)** cells. The cells were treated with different concentrations of CTU in the presence of RANKL. Total resorption pit area was measured and the results are shown as % of RANKL treatment. Pit formation assay of osteoclasts and quantification of pit area. *p < 0.05 compared with the only RANKL-treated group.

Because CTU inhibited the formation of osteoclasts and F-actin rings, we measured pit formation by inducing osteoclasts on a bone-biomimetic synthetic surface to investigate their effects on bone resorption. We found that CTU significantly inhibited the pit area in a concentration-dependent manner, and there was almost no pit area at the highest concentration (**Figures 4A, B**). Therefore, CTU inhibited osteoclast differentiation and function in bone resorption in RAW264.7 and BMM cells induced by RANKL.

### CTU Inhibits Osteoclast Migration by Regulating CCL4

**Figure 5:**
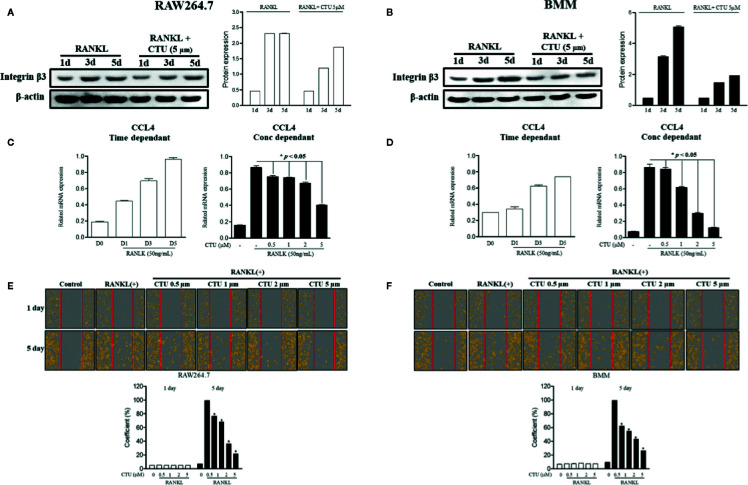
The effect of CTU on RANKL-induced osteoclast migration. RAW 264.7 and BMM cells were cultured in the presence of CTU and RANKL for 1–5 days to induce osteoclast differentiation, and the protein expression of integrin β3 was measured by Western blot **(A, B)**, and the levels of CCL4 mRNA were measured by real-time PCR **(C, D)**. RAW264.7 and BMM cells were cultured without or with RANKL for 3 d, and further incubated for 24 h in the presence of CTU (0.5, 1, 2, and 5 μM) after gentle scratching **(E, F)**. The number of migrated cells was averaged using IncuCyte-image marking software. *p < 0.05 compared with the only RANKL-treated group.

Chemokine CCL4 has been suggested to play an important role in osteoclast migration. Therefore, we measured the RNA expression of CCL4 in RANKL-treated RAW264.7 and BMM cells. As a result, the expression of CCL4 increased the most after 5 days of RANKL treatment. On day 5 of RANKL treatment, CTU inhibited CCL4 in a concentration-dependent manner (**Figures 5C, D**). CTU effectively inhibited the expression of integrin β3 in RANKL-treated RAW264.7 and BMM cells (**Figures 5A, B**). In addition, to measure the inhibitory effect of osteoclast migration on CTU, the coefficient of migrated cells was measured, as a result, the increased migration coefficient by RANKL on day 5 in RAW264.7 and BMM cells was reduced by concentration-dependent treatment of CTU (**Figures 5E, F**).

### CTU Inhibits the Expression of RANKL-Induced Osteoclast-Specific Genes

**Figure 6:**
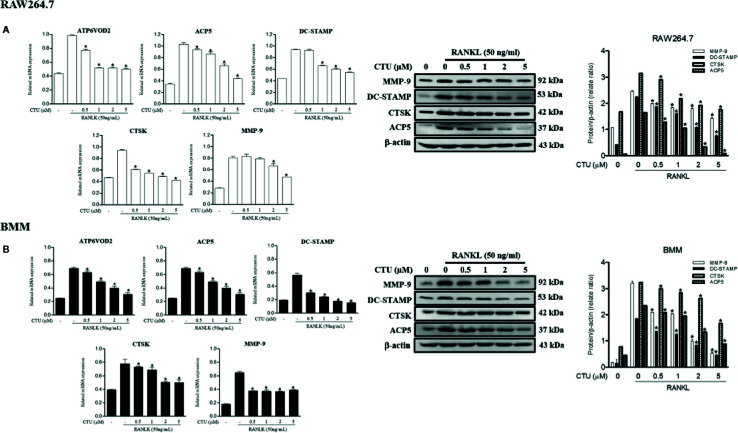
The effect of CTU on osteoclast-specific gene and protein expression in RANKL-induced RAW 264.7 **(A)** and BMM **(B)** cells. The cells were treated with different concentrations of CTU in the presence of RANKL, and osteoclast-specific gene expression was measured by real-time PCR. The results were normalized to GAPDH expression. *p < 0.05 compared with only RANKL-treated group.

The maturation of osteoclasts is accompanied by the expression of specific genes and protein, ATP6VOD2 and ACP5, DC-STAMP, CTSK and MMP-9, which are essential for osteoclast formation and function. Therefore, we investigated the role of specific genes and protein in osteoclast formation and function under the inhibitory effect of CTU on osteoclast differentiation and bone resorption. Treatment with CTU significantly down-regulated the expression of ATP6VOD2 and ACP5, DC-STAMP, CTSK, and MMP-9 more than in RANKL-treated group (**Figures 6A, B**). As a result, CTU suppressed differentiation and function by down-regulating the expression of osteoclast specific genes and proteins.

### CTU Suppress RANKL-Induced TRAF6-TAK1 Complex Formation

**Figure 7:**
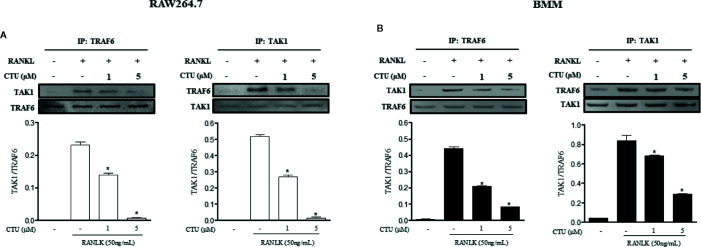
The effect of CTU on expression of TRAF6 and TAK1 in RANKL-induced RAW 264.7 and BMM cells. RAW 264.7 **(A)** and BMM **(B)** cells were pretreated with CTU for 2 h, and then treated with RANKL for 24 h. The cell extracts were subjected to western blot analysis. RAW264.7 **(A)** and BMM **(B)** cells were cultured for 3 days with RANKL (50 ng/ml), and pretreated with CTU. Cell lysates were immunoprecipitated (IP) with anti-TRAF6 or anti-TAK1 and immunoblotted with anti-TAK1 or anti-TRAF6, respectively. Expression of TRAF6 and TAK1 in cell lysates was measured by immunoblotting. The level of co-immunoprecipitated TAK1 or TRAF6 was quantified and normalized to total TRAF6 or TAK1.*p < 0.05 compared with the only RANKL-treated group.

TRAF6/TAK1 complex formation is an important step prior to RANKL-mediated MAPK and NF-κB activation. Therefore, we used co-precipitation to investigate the effects of CTU on the formation of RANKL-induced TRAF6/TAK1 complex. In pull-down assays, co-immunoprecipitation of TAK1 with anti-TRAF6 antibody (and of TRAF6 with anti-TAK1) was increased in the presence of RANKL. However, CTU inhibited co-precipitation of RANKL-induced TRAF6/TAK1 complexes in a concentration-dependent manner (**Figures 7A, B**).

### CTU Suppresses RANKL-Induced Activation of NF-κB and MAPKs

**Figure 8:**
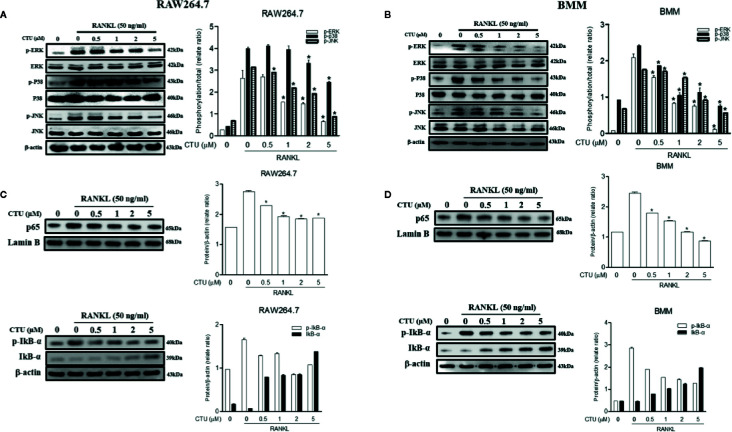
Effect of CTU on MAPKs **(A, B)** and NF-kB **(C, D)** activation in RANKL-induced RAW 264.7 and BMM cells. The cells were pre-incubated with or without CTU for 2 h, followed by treatment with 50 ng/ml RANKL for 30 min. The protein expression of cytosolic and nuclear p65, IкB¥á, and p-IкB¥á was compared with that of controls subjected to western blot analysis. *p < 0.05 compared with the only RANKL-treated group.

Downstream of TRAF6 signaling complexes, the activation of MAPK (ERK, JNK and p38 MAPK) and NF-κB plays an important role in osteoclast differentiation. Therefore, we used western blots to further explore the activation of the MAPKs and NF-κB pathways by which CTU regulates osteoclast differentiation and function. Phosphorylation of MAPKs and NF-κB p65 activity were induced in RANKL-stimulated RAW264.7 and BMM cells, and the expression of p-ERK, p-JNK, and p-p38 was inhibited by CTU (**Figures 8A, B**). CTU also inhibited the activity of p65 and the phosphorylation of IκB-α (**Figures 8C, D**). Thus, the osteoclast-differentiation and bone-resorption functions of CTU were related to MAPKs and NF-κB activity.

### CTU Downregulates RANKL-Induced Expression of NFATc1 and c-Fos

**Figure 9:**
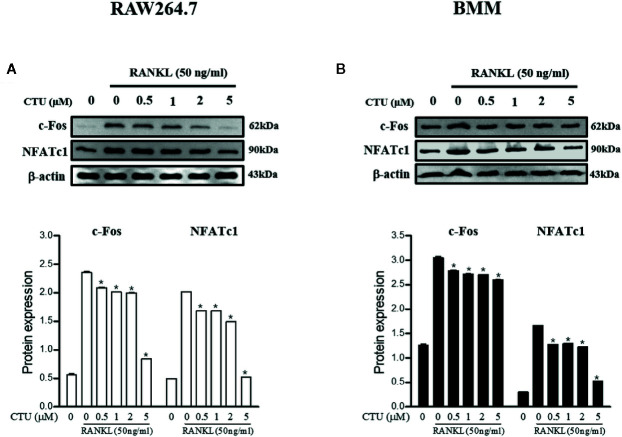
The effects of CTU on NFATc1 and c-Fos protein expression in RANKL-induced RAW 264.7 **(A)** and BMM **(B)** cells. The cells were cultured in the presence of RANKL with the CTU. After 24 h, the total protein was isolated and the protein expression levels were evaluated by western blots.*p < 0.05 compared with the only RANKL-treated group.

NFATc1 and c-Fos are the most important osteoclast-specific transcription factors following RANKL binding to RANK. Therefore, we used the western blot to evaluate the effects of CTU on the two transcription factors. RAW 264.7 and BMM cells induced by RANKL for 24 h significantly increased the expression of c-Fos and NFATc1, whereas in the group treated with CTU 0.5 and 1 μM in RAW264.7 and BMM cells, similar levels of NFATc1 and c-Fos expression were suppressed, and 2 and 5 μM treatment group showed a marked inhibitory effect (**Figures 9A, B**).

## Discussion

Disruption of bone homeostasis is caused by increased bone resorption by osteoclasts rather than new bone formation by osteoblasts, and RANKL is a major pro-osteoclastogenic cytokine that mediates osteoclast differentiation (Park et al., 2017; Ono and Nakashima, 2018). Thus, the inhibition of osteoclast formation, bone resorption and migration by inhibiting RANKL signaling and downstream pathways is an important target in the treatment of osteoporosis (Zou et al., 2001; Xuan et al., 2017). In the present study, we evaluated the mechanism of CTU inhibition of osteoclast differentiation and migration in a RANKL-induced RAW 264.7 and BMM cell line. Osteoclast differentiation by RANKL activates TRAP, a representative osteoclast marker involved in bone resorption, forms and maintains actin ring formation on the bone surface, and promotes bone resorption (Hayman, 2008). In our study, CTU attenuated osteoclast differentiation and function by inhibiting TRAP activity, F-actin formation, and bone resorption. Also, proteolytic enzymes, such as cathepsin K and MMP-9, and osteoclastogenesis-related markers, such as ACP5, DC-STAMP, and ATP6V0d2 play an important role in bone resorption and osteoclast differentiation. CTU inhibited these osteoclast gene markers in a concentration dependent manner.

In osteoclast signaling by RANKL, the cytoplasmic domain of RANK recruits TRAF6 to initiate complex formation with TAK1, activates NF-κB and MAPK, and plays a critical signaling role in osteoclast maturation (Mizukami et al., 2002; Nakashima et al., 2012). The TRAF family consists of six proteins each. TRAF6 is a unique member of the RING domain ubiquitin ligase family, which catalyzes the poly-ubiquitin chain linked *via* ubiquitin Lys-63 (Arch et al., 1998; Lamothe et al., 2007; Landstrom, 2010). In addition, the TRAF6 pathway induces phosphorylation of MAPKs, such as ERK, p38, and JNK, and regulates the expression of transcription factors such as NF-κB upon binding of RANKL and RANK in osteoclast precursor cells (Kobayashi et al., 2001). During RANKL signaling, MAPK is an important target for osteoclast differentiation as a major regulator of various cellular responses, including cell proliferation, apoptosis, and differentiation (Sui et al., 2014; Thummuri et al., 2015).We found that CTU inhibited the formation of TRAF6-TAK1 complex by RANKL stimulation in RAW264.7 and BMM cells and inhibited phosphorylation and transcription of NF-κB and phosphorylation of MAPK (JNK, ERK, p38) in a concentration-dependent manner. In addition, NFATc1 inhibition by CTU may be caused by down-regulation of c-Fos. Therefore, inhibition of the TRAF6-TAK1 complex formation plays an important role in osteoclast differentiation. In our immunoprecipitation assay, RANKL increased TRAF6 and TAK1 association, which was inhibited by CTU. These results suggest that CTU targets TRAF6-TAK1 complex formation to inhibit osteoclast differentiation.

RANKL regulates a variety of transcription factors, such as NF-κB, c-Fos, and NFATc1, and induces NFATc1 early to form mature and active osteoclasts (Teitelbaum and Ross, 2003). NFATc1 has been reported to regulate genes, such as TRAP, cathepsin K, and integrin β3 involved in osteoclast differentiation and function (Kim et al., 2014). In addition, osteoclast migration plays an important role in diseases associated with abnormal bone resorption such as rheumatoid arthritis and osteoporosis. In recent studies, chemokine CCL4 has been shown to mediate cell migration and bone invasion in RANKL-induced bone resorption (Xuan et al., 2017). In this study, CTU downregulated the expression of transcription factors and genes involved in RANKL-induced osteoclast differentiation and migration.

Recent studies investigating the osteoclast-differentiation inhibitory effects of natural compounds, such as flavonoids or polyphenols, have been actively conducted (Kim et al., 2018). However, prenylated xanthones have yet to be investigated for their role in inhibition of bone disease or osteoclast differentiation, despite their various antioxidant, anti-atherosclerotic, anti-inflammatory, and hepatoprotective activities (Lee et al., 2009; Jeong et al., 2010; Kim et al., 2019). Therefore, in this study, CTU isolated from *M. tricuspidata* suggests a potential therapeutic agent for osteoporosis by inhibiting proteins and specific genes that play an important role in the regulation of osteoclast differentiation and bone resorption and migration.

## Data Availability Statement

The original contributions presented in the study are included in the article/supplementary material; further inquiries can be directed to the corresponding author.

## Author Contributions

E-NK performed the experiments and wrote the manuscript. H-SL and JK performed the statistical analysis. G-SJ, DL, and SL participated in study design and coordination as well as drafting the manuscript. All authors contributed to the article and approved the submitted version.

## Funding

This research was supported by Basic Science Research Program through the National Research Foundation of Korea (NRF) funded by the Ministry of Education (NRF-2016R1A6A1A03011325).

## Conflict of Interest

The authors declare that the research was conducted in the absence of any commercial or financial relationships that could be construed as a potential conflict of interest.
